# Role of MRI-Based Functional Imaging in Improving the Therapeutic Index of Radiotherapy in Cancer Treatment

**DOI:** 10.3389/fonc.2021.645177

**Published:** 2021-08-27

**Authors:** Mei Li, Qin Zhang, Kaixuan Yang

**Affiliations:** ^1^Department of Gynecology and Obstetrics, Key Laboratory of Birth Defects and Related Diseases of Women and Children, Ministry of Education, West China Second University Hospital, Sichuan University, Chengdu, China; ^2^West China School of Medicine, West China Hospital, Sichuan University, Chengdu, China

**Keywords:** functional imaging, organs at risk, target volume, radiation therapy, therapeutic index

## Abstract

Advances in radiation technology, such as intensity-modulated radiation therapy (IMRT), have largely enabled a biological dose escalation of the target volume (TV) and reduce the dose to adjacent tissues or organs at risk (OARs). However, the risk of radiation-induced injury increases as more radiation dose utilized during radiation therapy (RT), which predominantly limits further increases in TV dose distribution and reduces the local control rate. Thus, the accurate target delineation is crucial. Recently, technological improvements for precise target delineation have obtained more attention in the field of RT. The addition of functional imaging to RT can provide a more accurate anatomy of the tumor and normal tissues (such as location and size), along with biological information that aids to optimize the therapeutic index (TI) of RT. In this review, we discuss the application of some common MRI-based functional imaging techniques in clinical practice. In addition, we summarize the main challenges and prospects of these imaging technologies, expecting more inspiring developments and more productive research paths in the near future.

## Introduction

**Figure 1:**
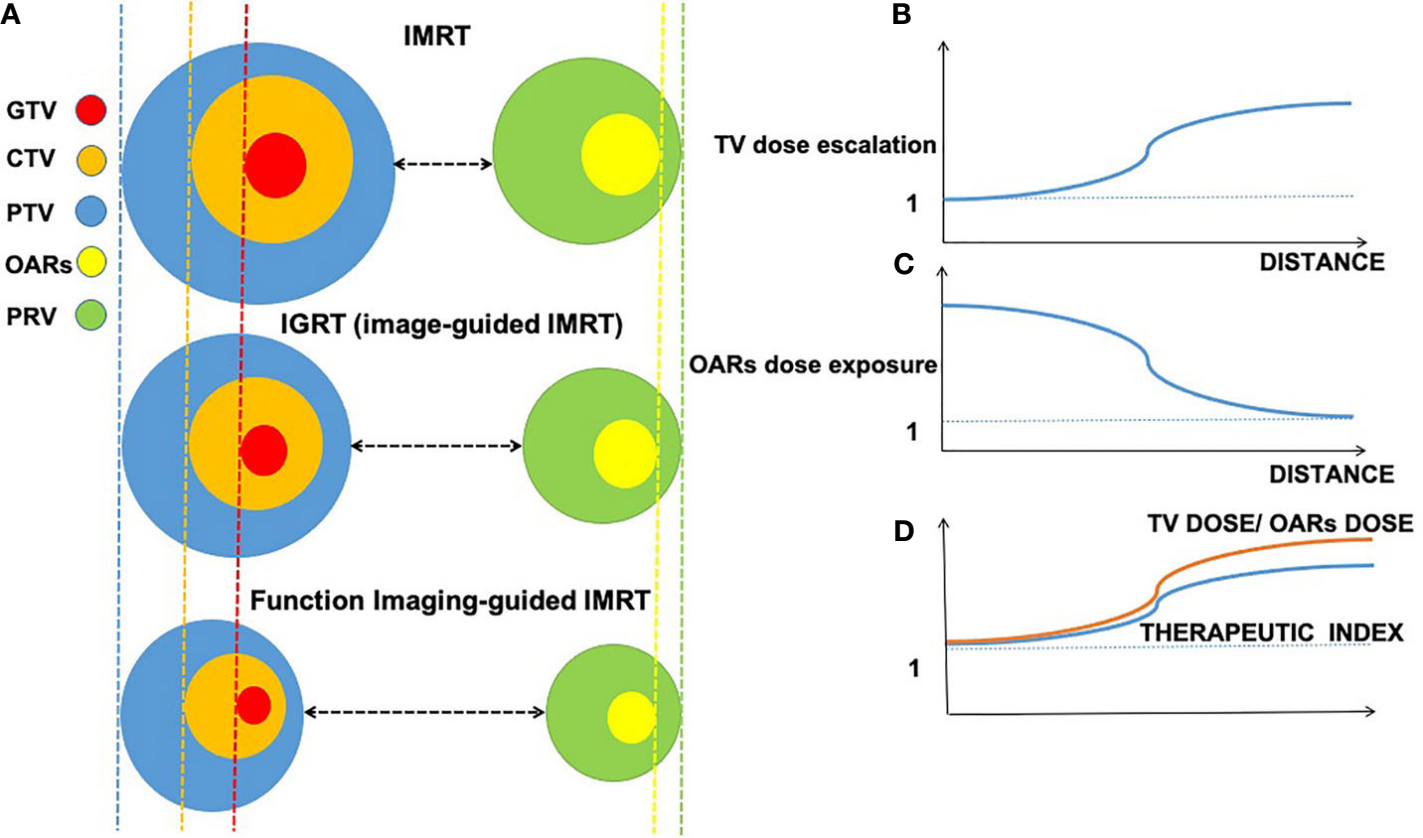
Interaction among imaging-guided techniques, treatment margins of tumor target, and organs at risk (OARs) based on the accuracy of imaging approaches. Decreasing volume around gross tumor volume (GTV, in red), clinical target volume (CTV, in orange), planning target volume (PTV, in blue), planning organ at risk volume (PRV, in green), and organs at risk (OARs, in yellow); distance changes between treatment margins and OARs in intensity-modulated radiation therapy (IMRT), image-guided radiation therapy (IGRT), and function imaging-guided IMRT. **(A)** Relations between distance (x-axis) of gross target and organ at risk. **(B)** Relations between the distance (x-axis) and TV dose escalation (y-axis). **(C)** Relations between the distance (x-axis) and OARs dose exposure (y-axis). **(D)** Relations between TV dose/OARs dose and therapeutic index.

Radiation therapy (RT) is the cornerstone of curative cancer care (1). Ideally, the optimal RT strategy is supposed to deliver the highest potential radiation dose to the tumor target volume (TV) without affecting nearby structures or organs at risk (OARs). To meet the requirement, radiation oncology has undergone a huge transition in the last decades from 2D therapy to 3D conformal RT (3DCRT) and intensity-modulated RT (IMRT), and even to intensity-modulated proton therapy (IMPT), which allows for better performance in terms of dose escalation in the TV and dose reduction in the OARs. Theoretically, therapeutic index (TI) is optimized by maximizing the dose in the TV while minimizing the dose in the OARs in the treatment plan (**Figure 1D**).

With regard to IMPT, it has been widely studied due to its unique depth–dose characteristics of protons, which can increase the dose to the TV while reducing the dose exposure to normal tissue, however, the majority of studies on the application of IMPT is based on small-scale studies and is poorly cost-effective (2). Therefore, herein, we focus on photon-based RT techniques such as 3DCRT and IMRT, which are currently the main RT strategies in large-scale clinical practice, despite the lack of TI optimization during RT. Fortunately, a growing body of evidence suggests that the integration of functional imaging techniques into RT brings hope for improving TI based on photon-based RT. In RT, imaging plays a critical role in tumor location, staging, target delineation, and outcome monitoring (3). In contrast to conventional IMRT and imaging-guided RT (IGRT), functional imaging-guided IMRT tends to provide a more accurate delineation of TV and OARs, resulting in diminishing treatment margin of the gross tumor volume (GTV), clinical target volume (CTV), and planning target volume (PTV) in tumor target and a smaller volume of OARs and planning OAR volume (PRV) in OARs (**Figure 1A**). In addition to anatomical information (such as location and size), functional imaging provides the physiological and functional status of the tumor and its surroundings compared with conventional magnetic resonance imaging (MRI) (3). Currently, functional imaging plays various roles in radiation oncology, such as tumor localization, staging, target delineation, assessment of early response to therapy, prognosis, and monitoring recurrence (4).

In general, functional imaging has widely enabled clinicians to improve TI ratios by reducing the risk of geographic miss and selectively increasing radiotherapy (RT) dose to the TV, and minimizing unnecessary dose exposure to the OARs simultaneously. In this review, we outline several common MRI-based functional imaging techniques that used in the field of RT to explore improvement in TI ratios. Furthermore, we summarize current challenges and prospects.

## Functional Imaging

At present, many functional imaging techniques have been gradually integrated into oncologic RT planning. Herein, we review some common imaging techniques in clinical practice, including diffusion-weighted (DW) imaging (DWI), intravoxel incoherent motion DWI (IVIM-DWI), MR-spectroscopic imaging (MRSI), dynamic susceptibility contrast (DSC), dynamic contrast-enhanced (DCE), diffusion tensor imaging (DTI)-MRI, and blood oxygenation level-dependent functional MRI (BOLD-fMRI) (**Table 1**).

**Table 1:** Different components of fMRI-based functional imaging.

Modality	Principle	Related parameters	Clinical utility in radiation therapy			
			Treatment preparation	Treatment planning	Treatment follow-up	Defects
DWI	Brownian motion of water molecule in biologic tissues (5)	DWI: ADC (6) IVIM-DWI: ADC, pure diffusion coefficient (D), pseudodiffusion coefficient (D*), perfusion fraction (f) (26–29).	DWI: tumor detection (7); staging (8–10); hypoxia (14, 15) IVIM-DWI: tumor detection (26, 30–33); benign and malignant identification (33–35); grading (28, 34, 36)	Delineation (8, 11–13); boost volumes (14, 15)	Treatment response (16–18); recurrent disease (19–24)	Poor geometric distortions; uncertainty of DWI-derived parameters (25)
MRSI	Biochemical and metabolic status of tissues (37, 38)	Cho, NAA, Lac, Cr (39–41)	Tumor identification (37, 42); diagnosis (41, 43–46); classification (39, 45, 47, 48); treatment planning (49, 50)	Delineation (49–51); boost volumes (49, 51–53)	Treatment response (37, 41, 54); recurrent disease (37, 55, 56)	Poor geometric distortions; uncertainty of diffusion parameters (57)
Perfusion MRI (DCE, DSC)	Tumor blood flow; Vascular permeability and vascularity (60–62)	DCE: ν e, K trans (58–60) DSC: BV and BF (73)	Tumor detection (61–64); treatment planning (17, 65); hypoxia (67, 68)	Tumor hypoxia (66–69); delineation (63, 64); dose painting (63)	Treatment response (17, 59, 61, 62, 65); recurrent disease (70)	Unstable CB V and CBF (71, 72)
DTI-MRI	anisotropic diffusion of water molecular in tissues (74)	ADC FA (74)	Tumor detection (75, 76)	Delineation (75–81); dose painting (75, 77, 79)	Treatment response (82, 83); recurrent disease (80, 81)	Image distortion (84)
BOLD fMRI	Cerebral oxygen utilization (85)	onset-time, time-to-peak, full-width-at-halfmaximum (FWHM) and amplitude (86)	positioning of the functional cortical center, including vision, movement, hearing, etc (87–93); hypoxia (94)	Trajectory delineation in surgery; delineation (95, 96)	Reduced surgical time (97)	Poor spatial resolution (98, 99); NVU (100, 101)

fMRI, functional magnetic resonance imaging; DWI, diffusion-weighted imaging; ADC, apparent diffusion coefficient; IVIM, intravoxel incoherent motion; MRSI, MR-Spectroscopic Imaging; Cho, choline; NAA, N-acetyl aspartate; Cr, creatine; DCE, dynamic contrast-enhanced; DSC, dynamic susceptibility contrast; BV, blood volume; BF, blood flow; CBV, cerebral blood volume; CBF, cerebral blood flow; DTI, diffusion tensor imaging; FA, fractional anisotropy; BOLD, blood oxygenation level dependent; NVU, neurovascular uncoupling.

*signal means false diffusion coefficient compared with true diffusion coefficient (D), mostly called pseudodiffusion coefficient (D*).

### Diffusion-Weighted MRI

DWI is a simple and readily available functional imaging technique with capability to visualize the motion of the water molecules (also called Brownian motion) in biological tissues (5). DWI can evaluate the differences in tissue cellular density and offer visibility of cellular construction (102, 103). Sensitized b-value, known as, the real diffusion weighting (measured in s/mm^2^), is used to measure the levels of diffusion weighting applied. Motion-sensitizing gradients are used for parameter acquisition. Thus, there is no need for contrast injection on DW-MRI due to its tissue contrast. The amount of diffusion (of water molecules) in different tissues can be quantificationally evaluated using the apparent diffusion coefficient (ADC) value (6) and then presented as a map, which holds better diffusion than traditional DWI. ADC value can be calculated by changing gradient amplitude using different b-values (104–106) and at least two b-values (107). In clinical practice, to achieve the lower mobility of water molecules and shorter diffusion distances in these tissues, using a larger b-value (e.g., b = 500 s/mm^2^) on DWI is suggested.

Generally, the spread of water molecules on DW-MRI is restricted on malignant lesions with hypercellular areas or complex structures, presenting a high signal on DWI and accompanying low ADC value (108, 109). Clinical practice shows (110) that the standard ADC value obtained by the traditional single-index DWI model is affected by the water molecule diffusion and microcirculation perfusion in the tissue and cannot well reflect the movement of water molecules in the tumor tissue. IVIM-DWI introduced by Le Bihan (5, 27) can more accurately assess diffusion movement and microcirculation perfusion in tumor tissues due to its ability to separate diffusion and perfusion. IVIM-based parameters involve pure diffusion coefficient (D), pseudodiffusion coefficient (D*), and perfusion fraction (f).

Based on the fact that the ADC value and signal intensity between tumor and normal tissue are significantly different, recently, DWI has been gradually explored to accurately delineate TV and OARs in radiation treatment planning. For example, it has been indicated that the addition of DWI to MRI can better distinguish lung cancer from atelectasis (7). DWI also takes advantage of nodal staging (8–10); thus, DWI provides smaller nodal TV delineation than conventional imaging (8). Similarly, DWI provides smaller rectal tumor volume (GTV) delineation than does T2-weighted (T2W) MRI (11–13), and probably because DWI provides a better edge contrast than T2W images (111). These findings indicate that a better RT boost to TV is possible. The quantitative data provided by DW-MRI could reflect intratumoral heterogeneity, highlighting more radioresistant areas (14, 15). Subsequently, elective dose escalation can be individually delivered to these areas. Moreover, DWI may be a potential tool to evaluate the salivary gland function in head and neck cancer (HNC) patients (16), resulting in the accurate protection for normal tissue, also with the ability to predict RT-induced xerostomia incident in RT. Several studies conclude that ADC histogram has a promising value of predicting overall survival (OS) and progression-free survival (PFS) and risk stratification in recurrent glioblastoma patients treated with bevacizumab (19–22).

Regarding clinical applications, IVIM-DWI can be used to detect positive resection margins in breast cancers (30, 31), lung tumors (26), and hepatic lesions in liver cancer (32, 33); and the diagnostic efficacy of D values is the highest (28, 29). A meta-analysis showed that the IVIM-DWI parameter (D value) showed better diagnostic performance than mono-exponential ADC (26). In addition, IVIM plays a pivotal role in benign and malignant identification (33, 34), especially for the D value (33). The D value is superior to ADC in distinguishing benign and malignant lesions (35). Moreover, IVIM-DWI-derived parameters can be used to grade malignant lesions (28, 34, 36) and have the potential to differentiate true progression from pseudoprogression after early chemoradiotherapy in glioblastoma multiforme (GBM) (23, 24). Besides, IVIM can predict treatment response, such as parotid changes and vertebral bone marrow changes (17, 18). Taken together, the ability of IVIM-DWI to detect and identify benign and malignant would be helpful in target delineation and OAR avoidance in RT. Regarding tumor grading, IVIM-DWI may aid in predicting tumor aggressiveness and prognosis. In terms of pseudo/true progression identification and prediction of treatment response, IVIM may help to determine early therapeutic intervention and improve prognosis.

Overall, DWI may be a promising tool to obtain a better TI when being incorporated into RT planning assistance. However, some limitations of DWI, such as geometric distortions, which are closely related to TV delineation, and uncertainty of diffusion parameters, where low reproducibility means high variations, impede its widespread use within clinical practice. To solve this issue, conjunction with other MR images is suggested such as higher-resolution anatomic images, high-quality data with different b-values, and, if possible, contrast material-enhanced images (25).

### MR-Spectroscopic Imaging

MRSI allows non-invasive measurement of biochemical information in tissues, especially in the brain with the existence of tumors. MRSI is also an analytical and non-injected contrast agent (CA) technique without ionizing radiation, related to MRI *in vivo* (37, 38). However, unlike MRI, which can only identify the anatomic structure and location of a tumor or normal tissue, MRSI can be used to determine the concentration and presence of various biochemical substances, often referred to as “metabolites” because of their role in metabolism. Therefore, MRSI can effectively supplement the characterization of tissue beyond MRI function. In recent years, proton MRSI has gained a great popularity due to its higher sensitivity and greater convenience, since proton MRSI does not need hardware modification while being performed on most MRI machines. Therefore, the remainder of this article in brain tumor studies focuses on protocols for ^1^H-MRSI. The common metabolites of ^1^H-MRSI include choline (Cho), *N*-acetyl aspartate (NAA), lactate (Lac), and creatine (Cr) in clinical routine (37).

In normal conditions, NAA exists in the intact neuronal and axonal structures, and its reduction demonstrates loss or damage of neuronal tissue (40). Cho is associated with phospholipid membrane turnover, and an increase in Cho indicates a process of leading to elevated glial proliferation and membrane synthesis (41). Lactate is implicated in various cancer mechanisms such as facilitating cancer cell proliferation and angiogenesis (112). Creatine is related to cellular energy metabolism. It is considered a useful reference metabolite due to its relative stability in different pathological processes involved in the central nervous system. In malignant tumors, NAA is reduced or lost since neurons are replaced by neoplastic tissue; Cho is increased directly; lactate may implicate a high level of malignancy (41). Based on the above, the NAA/Cho ratio is a sensitive marker for brain tumors, with the potential to distinguish active tumors from normal tissue and other non-cancerous lesions, such as necrosis (42). Cho/NAA ratio is widely utilized to describe tumor volume and invasion, because these metabolites change inversely in the tumor, increasing contrast (47). At present, the application of MRSI in clinical practice is involved in differential diagnosis (43–46), classification (45, 46), staging (46), treatment planning (49, 50), and posttreatment monitoring (54). Moreover, Lac/Cr, NAA/Cho, and Lac/NAA can predict overall survival (OS) and progression free survival (PFS) (41).

Herein, we mainly focus on the use of MRSI in radiation planning. In glioma, compared with conventional RT alone, adding of MRSI could provide metabolism information of tumor cells and OARs, resulting in more accurate target delineation (50). Croteau et al. conducted a study in 31 low- and high-grade-glioma patients. Their findings showed that MRSI can more accurately define the tumor boundary and normal tissues and can quantify the extent of the disease compared with conventional MRI *via* histopathological validation (54). Pirzkall et al. studied 34 patients with high-grade gliomas (52). When using T2 to define high-risk regions, the volume is extended by as much as 28 mm as compared with tumor definition from MRSI. Thus, the MRSI technique may have the potential to accurately optimize dose distribution of tumor TV and reduce the exposure to normal tissue (52). Moreover, Narayana et al. showed that Cho/Cr greater or equal to 3 defined by MRSI reduced 40% GTV volumes (GTVs) compared with GTVs defined by T1-weighted MRI (49). A study by Deviers et al. showed that tumor areas with lactate-to-*N*-acetyl aspartate ratio (LNR) 0.4 voxels before RT are likely to relapse, suggesting additional biological TVs for dose painting in GBM (53). In addition, a small study demonstrates that MRSI can aid in the delineation of hypoxic regions in solid tumors by exploring the metabolic outcome of tumor hypoxia, presenting increased total choline-containing metabolites (tCho) and lipid CH3 in breast tumors (51). Previous studies demonstrated that MRSI possesses great potential for the differentiation of tumor recurrence from radiation necrosis (37, 55, 56). This technology would be helpful for reirradiation settings in brain tumors due to its accurate delineation of recurrent lesions and successful avoidance of normal structures or radiation-induced reactions.

Overall, it seems that MRSI has been gradually and widely used to improve treatment planning for RT, with the ability to deliver dose escalation at particular tumor targets and reduce dose exposure to OARs. Even for recurrent disease, MRSI may perform well in improving TI, such as identifying and predicting tumor lesion recurrence. However, we should also be aware of the limitations of MRSI, such as the presence of spectral artifacts, which can result in false information, and lower specificity, although spectral artifacts may be solved by the automatic testing system (57) and by obtaining a higher field strength, like using 3T (113). A combination between MRSI and DW-MRI may improve the specificity of MRSI (114). However, other obstacles in MRSI, concerning low spatial resolution, long-time acquisition, and unstable acquisition of high-quality spectral images, have impeded the widespread translation of MRSI in clinical practice.

### Perfusion MRI

Perfusion MRI is a well-established perfusion imaging technique, mainly including three techniques: DSC, DCE, and arterial spin labeling (ASL) MRI. Based on perfusion MRI modality, different parameters could be calculated, such as blood volume (BV) and blood flow (BF) in DSC, also the transfer rate constant (K^trans^) and the extravascular extracellular volume fraction (Ve) value in DCE. In perfusion MRI, individuals under inspection need a gadolinium-based agent *via* a peripheral vein during the continuous imaging process.

DSC refers to the BF through a certain tissue in a unit of time, which is an important physiological characteristic of the tissue and can specifically reflect the characteristics of vascular lesions. In DSC, the post-processed acquired time series can be used to acquire perfusion maps with the above parameters. DCE can measure the hemodynamic properties of tissues, such as density, integrity, leakiness, and permeability of tissue vasculature, by obtaining continuous MRI from the pre- and post-intravenous injection of a CA (58, 61). To acquire quantitative DCE-MRI data, three main components of measurements are needed: 1) an original T1 map before contrast administration; 2) acquiring T1-weighted images after CA injection at a proper temporal resolution; and 3) a method to evaluate the arterial input function (59). Compared with DSC-MRI, T1 DCE-MRI has a lower temporal resolution and is mainly used to reflect the density of microvessels.

Based on all aforementioned data, perfusion MRI plays an essential role in tumor treatment within clinical practice. The formation of increasing new blood vessels is essential to the growth of malignancy (73); as a result, BV and BF will rise correspondingly. In brain tumors, previous studies have shown that cerebral BV (CBV) and cerebral BF (CBF) are associated with predicting clinical outcomes, such as OS and PFS (62). A study in high-grade-glioma and low-grade-glioma patients indicated that CBV (the mean relative CBV = 1.75) correlates positively with disease progression (62). However, in clinical practice, the use of median CBV and CBF is limited because of highly heterogenous gliomas, resulting in low sensitivity and specificity of assessing efficacy. Additionally, it should be noticed that a tumor may display low-rise CBV. An increasing number of studies indicate that DCE-MRI may improve differential diagnosis, localization, tissue features, staging, and monitoring treatment response in neoplastic diseases (60, 70, 115, 116). In prostate cancer, previous studies demonstrate that DCE-MRI may be useful for delineating both tumors and surrounding normal tissues in the prostate gland with sensitivity of 59%–87% and specificity of 74%–84% (63, 64, 117). DCE could predict the volume change of radiation-induced parotid by evaluating individual microvascular perfusion and tissue diffusion rates in HNC patients, suggesting an adjustment of the treatment plan before RT (17, 65). In advanced cervical carcinoma, evidence illustrates that DCE is likely to provide a clinically useful biomarker for the prognosis based on pharmacokinetic analysis of DCE-MRI data (118, 119). Additionally, different levels of tumor hypoxia are common during RT but are not easily assessable in patients. In clinical practice, parameter K^trans^ is a potentially useful biomarker for tumor hypoxia, RT resistance, and metastasis in cancers (66–69) as well as Ve (66, 69).

Although DSC and DCE have been relatively mature, there are still some limitations of parameter calculation in clinical practice. First, CBV and CBF are always changeable in any region of gliomas owing to circuitous vasculature and immature vascular structures, especially in high-grade gliomas (71, 72). Second, many factors may influence the leakiness of blood vessels, such as vascular permeability, BF, vascular lumen area, and even temperature (120–122). Therefore, K^trans^ and Ve value can be influenced easily by the above factors and not only reflect vessel permeability. A study conducted by Law et al. probably provides us with some insights into which a combination between DSC and DCE can significantly improve diagnostic accuracy and sensitivity (62).

### Blood Oxygenation Level-Dependent Functional MRI

BOLD-fMRI signals provide real-time cerebral oxygen distribution under normal physiological conditions, based on local magnetic field properties resulting from a mismatch between local oxygen consumption caused by neuronal activity and increased CBF reactivity (85). BOLD signal has four parameters: onset time, time to peak, full width at half maximum (FWHM), and amplitude (86). The increase of local CBF causes a reduction in the local amount of deoxygenated hemoglobin generated by metabolism in response to neuronal activity, presenting signal enhancement (T2 and T2*) in related brain regions (100). Among these regions, transparent contrast relies on repeatedly averaging subtle differences of signal enhancement. Brain activation mapping using BOLD-fMRI is based on the prerequisite that there is a tight coupling between neuronal activity and hemodynamic changes (101). Deoxyhemoglobin in the blood vessels is regarded as an endogenous CA during the production of functional activation maps. Oxyhemoglobin contains an unpaired electron and is therefore diamagnetic. Deoxyhemoglobin contains four unpaired electrons and is a paramagnetic substance. The different concentrations of oxyhemoglobin and deoxyhemoglobin will cause local magnetic field inhomogeneity, leading to the difference in the signal on the image and then production of imaging.

Current clinical application is mainly used for the positioning of the functional cortical center, including vision, movement, and hearing. BOLD-fMRI has become a valuable tool for presurgical functional brain mapping. In surgery, BOLD-fMRI can guide the safest surgical trajectory between functionally viable brain tissue and the lesion. In particular, in brain tumor patients, who are often neurologically impaired, we should take patient preparation and neurobehavioral evaluation into special consideration when performing fMRI. BOLD-fMRI for preoperative planning allows for accurate risk assessment of surgery-related brain damage, such as postoperative motion, visual and somatosensory deficits, and intraoperative cortical stimulation (ICS) mapping, reducing surgical time (97) and need for further preoperative diagnostic studies (123). Moreover, BOLD-fMRI demonstrates an excellent concordance with ICS for motor mapping (87–90) and language mapping (91–93). Wang et al. and Pantelis et al. have demonstrated that the combination of BOLD-fMRI and DTI could be beneficial of marking and sparing OARs in radiation treatment planning, resulting in less radiation toxicity (95, 96). In addition, a study showed that BOLD-fMRI is a non-invasive technique that could explore hypoxia information by analyzing the correlations of the *R value and HIF-1α (94).

Spatial resolution is one of the limitations of BOLD-fMRI, as flow increases in some of the larger arteries or veins feeding or draining large neuronal areas. The solution is to insert a “diffused” gradient pulse (corresponding to low b-values) into the MRI sequence; the largest vascular contribution in the BOLD signal (high D* values are associated with fast flow) can be squeezed to improve the spatial resolution of the activation map (98, 99). In addition, neurovascular uncoupling is another key limitation that could affect the accuracy of BOLD-fMRI surrounding brain tumors, but combining an observed vaso-task dependency with the BOLD signal analysis may partially overcome this shortcoming (101).

### Diffusion Tensor Imaging–MRI

DTI-MRI is an MRI technique that can be used *in vivo* non-invasively to measure anisotropic diffusion of water molecules in various tissues, leading to producing neural tract images (74). Abnormalities in the fiber structure of the axonal (white matter) can be detected by DTI-MRI, which can model brain connectivity. Thus, it has been rapidly developed to implement RT of white matter disorders (77). At present, DTI-MRI had been extensively utilized for glioma researches. DTI parameters include ADC and fractional anisotropy (FA) values (74).

Price et al. showed that glioma cells are prone to invade tissue along the direction of white matter tracks (WMTs) (75). Another study by Krishnan et al. calculated routes of water diffusion from the primary tumor location to tumor progression location by using DTI-MRI in glioma patients. Meanwhile, this study also showed that the direction of elevated water diffusion may be a reliable indicator of routes of tumor progression (78), which is consistent with Price’s study. Thus, DTI MRI would help localize and identify the possible microscopic disease and will help to delineate CTV more accurately. Also, some previous studies have proved that it may aid in the optimal delineation of biological CTV by incorporating DTI-MRI into RT planning while reducing the dose exposure to nearby function regions (76, 78–81). A study by Conti et al. demonstrated that radiation dose exposure to OARs was decreased by up to 16.86% after the integration of functional neuroimaging as compared with their initial values (124). In addition, DTI-MRI can evaluate response to neoadjuvant chemotherapy in patients with breast cancer (82, 83).

In DTI, there are still some other technique defects, such as the distortion of the image because of an uneven magnetic field and poor display for smaller fiber bundles (84). However, DTI has been rapidly developed in nervous system diseases, with an extensive clinical prospect to optimize TV, predict prognosis, and better protect functional regions of the brain.

## Therapeutic Index in Radiation Therapy

Conventional MRI already anatomically offers better soft tissue imaging in contrast to CT (125). However, it is not enough to provide high sensitivity and specificity delineation of TV due to a lack of functional indications. At the anatomic level, previous studies have demonstrated that functional imaging can provide a more accurate delineation of tumor TV base on better background contrast between tumor and normal tissue (12, 63, 64, 113, 117). Accurately defining the boundaries between tumor and normal tissue gives rise to the increased distance between tumor and OARs in function imaging-guided IMRT (**Figure 1A**). Moreover, the increased distance is beneficial for imaging-guided dose escalation in TV and decreased dose exposure in OARs (**Figures 1B, C**). In addition to the clear anatomical display, we note that there are several concepts and advanced radiation techniques related to fMRI-based RT that have great potential to improve TI, including tumor hypoxia, dose painting, adaptive RT (ART), local recurrence, and MR-guided linear accelerator (MR-LINAC). Among them, the integration of fMRI can provide TI benefits.

### Tumor Hypoxia

Despite improvement in the accuracy of target delineation and radiation delivery due to advances in RT and imaging techniques, in-field recurrence remains the predominant local failure model (126), which may be explained by the fact that the PTV of the entire primary tumor and the involved lymph nodes is delivered with a homogenous radiation dose without considering the intratumoral heterogeneity. Heterogeneity is one of the inherent characteristics of tumors, which is mainly manifested in the heterogeneity of intracellular and molecular biological features (including glucose metabolism, cell proliferation, hypoxia, epithelial growth factor receptor (EGFR) expression, and choline metabolism, etc.) (127). Tumor hypoxia is fairly common in RT due to abnormal vessel structures. Hypoxia is highly associated with poor prognosis because oxygen-deprived cells are strongly resistant to chemoradiotherapy, which leads to failure. Therefore, identifying and quantifying tumor hypoxia are important and necessary for improving TI in RT treatment. Numerous studies have shown that fMRI is an attractive option to identify, quantify, and spatially map hypoxic areas prior to therapy, as well as to track hypoxia changes during radiation (51, 66–69, 94), resulting in guiding increased radiation doses to hypoxic RT-resistant areas.

### Dose Painting

The concept of dose painting has been proposed to improve local control through increasing the dose to a segment of intra-tumor radiation-resistant while decreasing the dose to a radiosensitive segment based on fMRI (126). IMRT can deliver non-uniform dose distribution, but how to follow the region of interest (ROI) remains unknown. Therefore, biological/molecular information derived from fMRI may be a help to identify and track tumor hypoxia, proliferation, and other ROIs. The fMRI-based dose painting opens a new era called “biological conformality.” Previous studies showed that fMRI-guided imaging could help radiation dose boost in certain areas by means of dose painting in tumor target (128–131) while sparing OARs (128–132). According to the quantitative parameter maps from functional imaging (126, 133), the radiation dose can be spatially redistributed in the target area and OARs. The dose mapping seems to be more reasonable than surgery, a way of all-or-nothing therapy, as intratumoral heterogeneity is a natural feature. DCE-MRI can also be used for dose painting based on its ability to display microvasculature permeability and BF, which is associated with the tumor’s oxygenation (134–137). DWI can reflect areas with a higher tumor burden (138, 139) and with radiation resistance (14, 15). MRSI possesses the ability to monitor tumor metabolism (140, 141) and is also useful for dose painting.

### Adaptive Radiation Therapy

In clinical practice, the target for RT is dynamic. It changes over a time frame, including position, size, shape, and biology. ART strategies systematically track variations in targets and adjacent structures to timely inform treatment-plan modification during RT. Monitoring variations is necessary because a single pre-treatment plan is inadequate to reflect the actual dose distribution on the tumor and its surroundings during RT (142). This technology allows for increasing doses delivered to tumors while simultaneously limiting dose exposure to the normal structures. MRI-guided ART provided both personalized geometric and biological adaption (143). Bladder cancer is well suitable for ART since the bladder has large inter-fractional changes and intra-fractional motion (144, 145). MRI-guided adaptive brachytherapy in cervical cancer (146, 147) and primary vaginal cancer (148) results in effective and stable long-term local control at all stages of cervical cancer, while decreasing severe radiation-induced morbidity. Similarly, promising results are also observed in liver tumors (149), prostate cancer (150), and unresectable primary malignancies of the abdomen. It seems like MRI-guided ART is more suitable for highly radiosensitive tumors with large motion changes and volume changes over the course of RT.

### Local Recurrence

Despite advances in RT and imaging techniques, in-field recurrence remains a common failure model. Recurrent lesions are a treatment challenge because of RT resistance and radiation dose limitation to surrounding normal tissues. The ability of fMRI to differentiate tumor recurrence to post-radiation treatment effects, including pseudoprogression and radiation necrosis, provides assistance in reirradiation settings in patients treated after primary chemoradiotherapy (23, 24). ADC values for radiation necrosis were found to be higher than for recurrence in some studies; however, the reported values are inconsistent and may be explained by technical factors (151). A study by Xu et al. (152) using DTI showed that the mean ADC ratios for radiation necrosis in recurrent tumors were 1.62 and 1.34. An alternative to DTI is that it favors tumor recurrence over radiation necrosis when diffusion restriction is present on the ADC image, which does not show diffusion restriction. DCE-MRI perfusion measures CBV and CBF, which can be accurately measured in the presence of a blood–brain barrier breakdown (151). A study by Larsen et al. (153) measured CBV in patients with MRI contrast-enhanced. They found that lesion regression shows low CBV (less than 1.7 ml/100 g), while lesion progression shows high CBV (>2.2 ml/100 g). Therefore, Larsen et al. concluded that an absolute CBV threshold of 2.0 ml/100 g can detect lesion degeneration or progression, with a reported sensitivity and specificity of 100%. Several studies have shown that patients with tumor recurrence have higher Cho/Cr and Cho/NAA values than patients with radiation necrosis (151, 154). In addition, fMRI can predict tumor aggressiveness and early treatment response, with the assistance of deciding early therapy intervention and improving the prognosis (17, 23, 24).

### MR-Guided Linear Accelerator

In recent years, with the advancement of MR-guided RT, accurate radiation is facing new challenges and prospects. MR-LINAC is a new technology and the first machine in the world to combine radiation equipment (such as a linear accelerator or ^60^Co sources) and high-resolution MRI (155). The key benefits of MR-LINAC are that it can record MR images for every fraction and use these for daily plan adaptation. During the radiation treatment of the tumor, the radiation beams are accurately located in and destroy tumors, while avoid radiating and harming the nearby healthy tissues. Currently, MR-LINAC has been applied in various tumors, including pancreatic cancer, prostate cancer, and liver cancer (156–159). To date, MR-LINAC seems to be the most efficient method to optimize the TI of RT in cancer treatment. Combining MRI with the most accurate RT techniques, such as proton therapy, will be our ultimate goal (160). However, prolonged treatment times, patient tolerance, and high cost are the major obstacles. At present, related studies on MR-LINAC are still at an early stage with sparse clinical evidence, despite interesting potential.

## Challenges and Prospects

Although the use of quantitative imaging was studied since the early 1990s in RT (125), there are still significant barriers to its widespread use in clinical practice. Excitingly, with the development of fMRI techniques, some prospects are inspiring.

### Challenges of Functional MRI for Radiation Therapy

From the object technology perspective, inconsistent imaging is commonly produced by different vendors, machines, and imaging protocols. However, quantitative imaging in RT is not a simple substitute for quantitative imaging in radiology. For example, in an RT scan, the patient’s position needs to be as similar as possible to the treatment setting. However, it is not easy to accomplish this in many institutions because of various policies and management. In addition, robust calibration for biomarkers is still a big challenge, as the current quantitative imaging technique needs one or multiple measurable parameters to produce a voxel, leading to in worse signal-to-noise ratio (SNR) and greater voxels than traditional imaging (107). Last but not least, when applying different imaging techniques together, it is difficult for clinicians to interpret multiparametric functional images with transparently conflicting information; for example, the overlap is largely restricted in the area identified by DWI and DCE-MRI simultaneously.

On the artificial perspective, currently, delineation of most tumors and OARs are still through manual procedures, in which the TV is determined by a radiation oncologist and a radiologist together on the basis of clinical information and images. Therefore, differences in which different spectators have various definitions in anatomic features of OAR boundary may cause inconsistency in the OAR definition. Moreover, errors caused by mistakenly missing or adding parts can give rise to inaccurate delineation in OARs. Finally, a small part of oncologists, particularly those in rural areas, do not receive sufficient training and clear instructions which leads to discrepancies in the anatomic definition of OARs.

From the economic perspective, compared with conventional MRI, fMRI is more expensive. This is because the application of fMRI requires higher machine costs. Some studies speculate that models combined with multiparametric MRI (mpMRI) have advantages of cost-effectiveness, but their findings are based on several *a priori* assumptions for every model (161–165). Therefore, any change in the selected criteria might result in a major change in the cost and, consequently, in the conclusions. Notably, to date, the evidence for the use of mpMRI to consider further biopsy is at best based on minimal clinical data (166, 167). Furthermore, such a policy is still under observation, as almost all publications are related only to systematic biopsy. There are several limitations associated with the widespread utilization of mpMRI in clinical practice. First, low-quality mpMRI is still a major issue. Surprisingly, the initial cost of repeat low-quality mpMRI is very common but not mentioned. Most importantly, there are no standardized principles to evaluate mpMRI progression in patients on AS. For example, how should we assess the lesion status in Prostate Imaging-Reporting? Is it an increase in tumor size, if so, how about the threshold? Finally, above all, evidence is mainly from small studies and extrapolation of various strategies such as enlarging the merit of initial mpMRI for biopsy.

### Prospects of Functional MRI for Radiation Therapy

According to the above challenges, there are some relative solutions. To acquire consistent and continuous images, we suggest that it would be better if patients are willing to receive a complete examination under same conditions. To analyze the conflicting information, some researchers suggest defining the area identified by two imaging approaches as gross tumor volume (GTV); the areas indicated by only one of the imaging techniques can be defined as CTV with high risk. To reduce inter-observer variability of OAR delineation, an automatically delineated system might be recommended in the future. Also, standardized training should be publicized widely.

Concerning cost-effectiveness improvement, the growing body of evidence demonstrates that fMRI may bring cost-effectiveness in prostate cancer. In contrast to transrectal ultrasound-guided biopsy (TRUS) alone, the use of mpMRI to select patients for repeat biopsy holds fewer biopsies and lower cost, despite that a few cancers are being missed, but further research is needed to determine whether missed cancers represent clinically significant tumors (168). Another study showed that an mpMRI-first strategy and then TRUS are cost-effective to diagnose clinically significant prostate cancer (169). In patients with low-risk cancer, overtreatment will lead to adverse effects and unnecessary costs (170). Active surveillance (AS) program, which contains digital rectal examination (DRE), prostate-specific antigen (PSA), and standard repeated 12-core TRUS, is currently recommended for avoiding overtreatment (170). However, the standard AS has some limitations, including missing high-risk tumors and performing unnecessary biopsies. The strategy of using mpMRI combined with limited MR-guided TRUS can improve quality of life and greatly reduce cost in low-risk prostate cancer patients during follow-up (170). Adequate data are further needed from large randomized prospective cohorts in the future.

In terms of some new insights or techniques, based on the above, the application of fMRI to guide dose painting and ART to detect tumor hypoxia area and postoperative local recurrence, in combination with MR-LINAC, has led to some promising findings. Despite the lack of a uniform measurement standard, we expect to further explore the function of fMRI in improving TI through large-scale randomized clinical studies.

## Conclusion

Incorporating functional imaging techniques into RT planning has big potential to improve TI in RT *via* different mechanisms. Functional imaging possesses potential advantages, as follows. First, quantitative imaging may provide superior contour and visibility between tumors and normal tissues, with the benefit of dose escalation in TV and dose reduction in OARs, with the promising potential for guiding de-escalation in oropharyngeal carcinoma patients (171, 172). Second, quantitative imaging may be a potent toxicity prediction tool during RT. These can give us a clinical indication for precaution. Third, the combination of different functional imaging techniques may make up for shortcomings that exist in single imaging. We believe that it is worthwhile to overcome the above challenges and explore larger, multicenter, randomized clinical studies on quantitative imaging in RT.

## Author Contributions

KY and ML: study design. KY, ML, and QZ: data collection. KY and ML: data interpretation and writing. All authors contributed to the article and approved the submitted version.

## Conflict of Interest

The authors declare that the research was conducted in the absence of any commercial or financial relationships that could be construed as a potential conflict of interest.

## Publisher’s Note

All claims expressed in this article are solely those of the authors and do not necessarily represent those of their affiliated organizations, or those of the publisher, the editors and the reviewers. Any product that may be evaluated in this article, or claim that may be made by its manufacturer, is not guaranteed or endorsed by the publisher.
